# Racial and Ethnic Disparities in Buprenorphine and Extended-Release Naltrexone Filled Prescriptions During the COVID-19 Pandemic

**DOI:** 10.1001/jamanetworkopen.2022.14765

**Published:** 2022-06-01

**Authors:** Thuy Nguyen, Engy Ziedan, Kosali Simon, Jennifer Miles, Stephen Crystal, Hillary Samples, Sumedha Gupta

**Affiliations:** 1 Department of Health Management and Policy, School of Public Health, University of Michigan, Ann Arbor; 2 Department of Economics, Tulane University, New Orleans, Louisiana; 3 O'Neil School of Public and Environmental Affairs, Indiana University, Bloomington; 4 National Bureau of Economic Research, Cambridge, Massachusetts; 5 School of Social Work, Rutgers University, New Brunswick, New Jersey; 6 Center for Health Services Research, Institute for Health, School of Social Work, Rutgers University, New Brunswick, New Jersey; 7 School of Public Health, Rutgers University, New Brunswick, New Jersey; 8 Rutgers Institute for Health, Health Care Policy and Aging Research, Rutgers University, New Brunswick, New Jersey; 9 Department of Health Behavior, Society and Policy, Rutgers School of Public Health Rutgers University, New Brunswick, New Jersey; 10 Department of Economics, Indiana University-Purdue University Indianapolis, Indianapolis

## Abstract

**Importance:**

COVID-19 disrupted delivery of buprenorphine and naltrexone treatment for opioid use disorder (OUD), and during the pandemic, members of racial and ethnic minority groups experienced increased COVID-19 and opioid overdose risks compared with White individuals. However, whether filled buprenorphine and naltrexone prescriptions varied across racial and ethnic groups during the COVID-19 pandemic remains unknown.

**Objective:**

To investigate whether disruptions in filled buprenorphine and naltrexone prescriptions differed by race and ethnicity and insurance status or payer type.

**Design, Setting, and Participants:**

This cross-sectional study used retail pharmacy claims from May 2019 to June 2021 from the Symphony Health database, which includes 92% of US retail pharmacy claims, with race and ethnicity data spanning all insurance status and payer categories. Interrupted time series were used to estimate levels and trends of dispensed buprenorphine and naltrexone prescriptions before and after pandemic onset. Included individuals were those who filled buprenorphine and extended-release naltrexone prescriptions. Data were analyzed from July 2021 through March 2022.

**Main Outcomes and Measures:**

Weekly rates of dispensed buprenorphine and extended-release naltrexone prescription fills per 1000 patients and proportion of longer (ie, ≥14 days’ supply) buprenorphine prescription fills were calculated. Analyses were stratified by patient race and ethnicity and further by insurance status and payer type for White and Black patients.

**Results:**

A total of 1 556 860 individuals who filled buprenorphine prescriptions (4359 Asian [0.3%], 94 657 Black [6.1%], 55 369 Hispanic [3.6%], and 664 779 White [42.7%]) and 127 506 individuals who filled extended-release naltrexone prescriptions (344 Asian [0.3%], 8186 Black [6.4%], 5343 Hispanic [4.2%], and 53 068 White [41.6%]) from May 6, 2019, to June 5, 2021, were analyzed. Prepandemic increases in buprenorphine fill rate flattened for all groups after COVID-19 onset (30.5 percentage point difference in trend; *P* < .001) compared with prepandemic trends. Significant level decreases in buprenorphine fills (ranging from 2.5% for Black patients; *P* = .009 to 4.0% for Hispanic patients; *P* = .009) at pandemic onset were observed for members of racial and ethnic minority groups but not White patients. At pandemic onset, rate of buprenorphine fills decreased in level for Medicare and cash-paying patients but with greater decreases for Black patients (Medicare: 10.0%; *P* < .001; cash: 20.0%; *P* < .001) than White patients (Medicare: 3.5%; *P* = .004; cash: 15.0%; *P* < .001). No decreases were found among Medicaid patients. Unlike buprenorphine, extended-release naltrexone had uniform level (from 10.0% for White patients with private insurance; *P* < .001 to 23.3% for Black patients with Medicare; *P* < .001) and trend (from 15.5 percentage points for White patients with Medicaid; *P* = .001 to 52.0 percentage points for Black patients with private insurance; *P* < .001) decreases across groups.

**Conclusions and Relevance:**

This study found that the COVID-19 pandemic was associated with immediate decreases in filled buprenorphine prescriptions by members of racial and ethnic minority groups but not White individuals. These findings suggest that members of racial and ethnic minority groups had larger losses in buprenorphine access during the pandemic across payer types.

## Key Points

**Question:**

Did pandemic disruptions in filled buprenorphine and extended-release naltrexone prescriptions for opioid use disorder (OUD) differ by race and ethnicity or insurance status and payer type?

**Findings:**

In this cross-sectional study of 92% of US retail pharmacy claims of buprenorphine among 1 556 860 individuals and extended-release naltrexone among 127 506 individuals for OUD from May 2019 to June 2021, prepandemic growth rate of buprenorphine fills significantly flattened overall after pandemic onset (30.5 percentage points relative trend decrease over 1 year). However, immediate significant level decreases in buprenorphine prescriptions at pandemic onset (2.5% to 4.0%) were concentrated among members of racial and ethnic minority groups but not White patients.

**Meaning:**

These findings suggest that the COVID-19 pandemic may have been associated with worsened disparities in filled buprenorphine and naltrexone prescriptions for OUD among members of racial and ethnic minority groups compared with White patients.

## Teaser

This cross-sectional study investigates disparities by race and ethnicity in disruptions to filled buprenorphine and naltrexone prescriptions for opioid use disorder associated with the COVID-19 pandemic.

## Introduction

COVID-19 deaths^1^ and increases in opioid overdose and death^2,3^ have disproportionately impacted racial and ethnic minority groups,^4,5^ intensifying pre-pandemic disparities.^6^ Methadone, buprenorphine, and naltrexone are Food and Drug Administration–approved medications for opioid use disorder (MOUD).^7,8^ Methadone is dispensed only in federal opioid treatment programs. Extended-release (XR) naltrexone (Vivitrol) has been increasingly used for opioid use disorder (OUD) treatment, particularly in criminal justice systems.^9^ Expanded use of buprenorphine is a policy priority given strong data on efficacy and potential provision in primary care.^10^

Despite some progress in MOUD use, prepandemic evidence suggested that it was associated with frequent exposure to high out-of-pocket costs^11^ and inequitable distribution, with greater access for White individuals^12,13,14^ and communities with primarily White populations.^15,16^ Even accounting for substantial insurance coverage differences by racial and ethnic group, buprenorphine and XR naltrexone disparities exist in privately^17^ and publicly^18^ insured populations.

The COVID-19 national emergency declaration in mid-March 2020 was associated with disruptions to the initiation and continuity of buprenorphine treatment.^19,20^ Racial and ethnic minority groups experienced higher rates of COVID-19 morbidity and mortality,^21,22^ and increasing evidence suggests that these groups had significantly lower prepandemic rates of buprenorphine prescribing.^23^ However, it remains unclear whether pandemic disruptions of these treatments were also concentrated among racial and ethnic minority groups. Trends beyond the initial acute months of the pandemic are also important, but prior studies^19,20,24^ of MOUD prescribing during COVID-19 focused on limited time frames and samples.

To reduce COVID-19 transmission, the federal government relaxed MOUD prescribing and dispensing policies by temporarily waiving certain requirements for in-person evaluations and visits. States were given greater Medicaid flexibility to mitigate care access and delivery challenges. However, substantial gaps remain in understanding how effectively these changes offset outcomes associated with pandemic disruptions in buprenorphine and naltrexone pharmacy dispensing and differential changes across racial and ethnic populations.

Our goal was to investigate the association of the onset of pandemic with immediate and longer-term changes in buprenorphine and XR naltrexone pharmacy dispensing for OUD. We investigated these changes during the 15 months after the March 2020 onset of the pandemic. We also aimed to investigate differential outcomes by race and ethnicity and payer type.

## Methods

This cross-sectional study, because it used fully deidentified data, was granted not regulated status and exemption from informed consent by the University of Michigan Medical School Institutional Review Board. The study followed the Strengthening the Reporting of Observational Studies in Epidemiology (STROBE) reporting guideline.

### Data Source and Sample

We analyzed retail pharmacy claims from May 2019 to June 2021 using the Symphony Health database, which captures 92% of total US retail pharmacy claims, 71% of mail-order pharmacy claims, and 65% of specialty pharmacy claims. The Symphony Health data were accessed through academic collaboration with the COVID-19 Research Database.^34^ The database records medication data for more than 280 million unique patients from more than 1.8 million prescribers, categorized by payment source.^25,26^ These data have been previously used to examine short-term changes in overall buprenorphine and naloxone prescriptions in the early pandemic months, as well as racial and ethnic disparities in diagnoses and medication dispensing for HIV.^19,24,27,28^ Our sample included 1 556 860 and 127 506 unique individuals who filled buprenorphine and XR naltrexone prescriptions, respectively, during May 6, 2019, to June 5, 2021.

### Measures

We classified race and ethnicity information into 5 categories: Asian, Black, Hispanic, White, and other or unknown. Symphony Health is an integrated health care data repository, and an individual’s race or ethnicity reflect clinician-reported information on patient health records from multiple sources, including electronic health records and medical claims obtained by Symphony Health. Race or ethnicity categories were not provided separately in the database, and we used combined race and ethnicity categorizations of the database as reported to Symphony Health. Hispanic ethnicity was reported without race, and individuals in the sample with reported race or ethnicity were assigned a single category. Individuals whose race and ethnicity were unknown were included in the national trend data analysis; however, they were excluded from race and ethnicity–specific analyses.

We examined national trends in total US prescription dispensing. Sales of buprenorphine and XR naltrexone were identified using National Drug Codes.^29^ We constructed 3 weekly prescribing measures: patients per 1000 patients with buprenorphine or XR naltrexone prescription fills among individuals with any prescription drug claim in the week and the proportion of longer-running prescription fills (ie, with ≥14 days of supply) among all dispensed buprenorphine prescription fills in the week. Because XR naltrexone is a long-acting injectable formulation, examining changes in the length of prescriptions was limited to buprenorphine.

We examined buprenorphine and XR naltrexone prescribing in the periods before (preperiod) and after (postperiod) the transition week (ie, March 16-22, 2020) starting immediately after the COVID-19 national emergency declaration. Following prior work,^19^ we defined the transition between these periods as occurring on March 16, 2020, and excluded March 8 to March 15, 2020. We conducted sensitivity analyses to ensure results were not sensitive to this exclusion.

All weekly prescribing outcomes were further examined in samples stratified by patient race and ethnicity (ie, Asian, Black, Hispanic, or White). They were then further stratified for White and Black patient populations by payer type (ie, Medicaid, Medicare Part D, commercial insurance, and cash-paid prescriptions). While the main analysis included all race and ethnicity categories, for the secondary analysis with further stratification by payer type, we focused on Black and White populations to reduce data dimensions sufficiently while being able to fully discuss results for all outcomes. Moreover, the overdose mortality rate for Black individuals surpassed that of White individuals in 2020 for the first time since 1999.^35^ We believe that by providing the first analysis of racial trends in MOUD access during the pandemic focusing on differences between Black and White patients may provide a baseline and inspire further research that may extend these results to other racial groups.

### Statistical Analysis

We examined the association between COVID-19 onset and buprenorphine or XR naltrexone prescribing with interrupted time series models estimating regression-adjusted changes in levels and trends in the preperiod and postperiod for each outcome. Models included a weekly linear time trend capturing the weeks since pandemic onset, an indicator for the preperiod or post period, and the interaction of the preperiod or postperiod indicator with the weekly linear time trend. Statistically significant preperiod and postperiod indicators would show immediate shifts in prescribing outcomes. Similarly, statistically significant coefficient estimates on the interaction of the preperiod or postperiod indicator with the weekly linear time trend would indicate significant changes in prescribing trends relative to the preperiod. All models were estimated using 101 study weeks (ie, May 2019 to June 2021). We examined raw trends in buprenorphine and XR naltrexone prescribing outcomes for the full sample and separately in subsamples with complete and missing race and ethnicity information. We plotted regression-adjusted weekly buprenorphine or XR naltrexone prescription fills by patient race and ethnicity and further stratified by insurance status within racial and ethnic groups. Data analysis was conducted with Stata statistical software version 17.0 (StataCorp) using 2-sided *t* tests for regressions, with significance set at *P* < .05. Data were analyzed from July 2021 through March 2022.

## Results

Our sample included 1 556 860 unique individuals who filled buprenorphine prescriptions and 127 506 unique individuals who filled XR naltrexone prescriptions. Among individuals who filled buprenorphine prescriptions, there were 4359 Asian individuals (0.3%), 94 657 Black individuals (6.1%), 55 369 Hispanic individuals (3.6%), 664 779 White individuals (42.7%), and 737 952 individuals (47.4%) with unspecified race or ethnicity (ie, unknown or other), while among individuals who filled XR naltrexone prescriptions, there were 344 Asian individuals (0.3%), 8186 Black individuals (6.4%), 5343 Hispanic individuals (4.2%), 53 068 White individuals (41.6%), and 59 290 individuals with unspecified race or ethnicity (46.5%). Among patients with buprenorphine prescriptions, 613 869 individuals (39.4%) had Medicaid, 197 728 individuals (12.7%) had Medicare, 435 731 individuals (28.0%) had private or commercial plans, and 254 093 individuals (16.3%) used cash payments, while among individuals with XR naltrexone prescriptions, 70 289 individuals (55.1%) had Medicaid, 11 681 individuals (9.2%) had Medicare, 37 643 individuals (29.5%) had private or commercial plans, and 5097 individuals (4.0%) used cash payments. Shares of patients filling buprenorphine and XR naltrexone prescriptions did not change significantly by patient race or ethnicity or payer type throughout the study period (eTable in the Supplement). The study sample share with unspecified race and ethnicity remained stable, at approximately one-half, throughout the study period (eTable in the Supplement).

### Changes in Buprenorphine and XR Naltrexone Prescribing After Onset of the COVID-19 Pandemic

Prior to the onset of the pandemic (ie, before the week of March 16-22, 2020), the estimated weekly number of individuals filling prescriptions per 1000 individuals (ie, intercept) was 0.7 individuals (95% CI, 0.7-0.8 individuals) for buprenorphine prescriptions and 0.02 (95% CI, 0.02-0.02 individuals) individuals for XR naltrexone prescriptions (Table 1). Prepandemic estimated weekly prescriptions of buprenorphine and XR naltrexone dispensed per 1000 individuals were higher among White individuals (buprenorphine: 1.1 individuals [95% CI, 1.1-1.2 individuals]; XR naltrexone: 0.03 individuals [95% CI, 0.02-0.03 individuals]) than among members of racial and ethnic minority groups (eg, buprenorphine: 0.2 individuals [95% CI, 0.2-0.2 individuals] among Asian patients, 0.8 individuals [95% CI, 0.7-0.8 individuals] among Black patients, and 0.5 individuals [95% CI, 0.5-0.5 individuals] among Hispanic patients). Prepandemic level and growth rate of buprenorphine dispensing were substantially larger than for XR naltrexone (Table 1). The prepandemic weekly rate of patients with filled prescriptions was trending upward for buprenorphine and XR naltrexone (Figure). To contextualize the size of weekly trends (slope), we calculated the implied 1-year growth relative to the baseline rate by multiplying the estimated weekly trend by 52 and dividing by the prepandemic intercept (which corresponds to the estimated level in March 16-22, 2020). Therefore, for buprenorphine, the prepandemic annual growth rates implied by the estimated trend (slope) were higher for White patients (28.4%) and Hispanic patients (31.2%) compared with Black patients (26.0%).

**Table 1.** Changes in Estimated Weekly Prescription Fills^a^

Variable	Patients with fills, No. (95% CI), patients/1000 patients/wk				
	Total	White	Hispanic	Black	Asian
**Buprenorphine**					
Estimated intercept^b^					
Prepandemic^c^	0.7 (0.7 to 0.8)	1.1 (1.1 to 1.2)	0.5 (0.5 to 0.5)	0.8 (0.7 to 0.8)	0.2 (0.2 to 0.2)
Pandemic^d^	0.7 (0.7 to 0.7)	1.1 (1.1 to 1.1)	0.5 (0.5 to 0.5)	0.7 (0.7 to 0.7)	0.2 (0.2 to 0.2)
Before-to-after change in level (%)^e^^,^^f^	−0.01 (−1.4)	−0.02 (−1.8)	−0.02 (−4.0)	−0.02 (−2.5)	−0.008 (−4.0)
*P* value	.17	.15	.009	.009	.04
Estimated weekly trend (slope)					
Prepandemic^c^	0.004 (0.003 to 0.004)	0.006 (0.005 to 0.006)	0.003 (0.002 to 0.003)	0.004 (0.004 to 0.005)	0.001 (0.0009 to 0.001)
Pandemic^d^	−0.0001 (−0.0004 to 0.0001)	−0.0003 (−0.0007 to 0.0002)	−0.00002 (−0.0002 to 0.0002)	−0.00005 (−0.0003 to 0.0002)	−0.00005 (−0.0002 to 0.00008)
Before-to-after change in trend (ie, slope)	−0.004	−0.006	−0.003	−0.004	−0.001
*P* value	<.001	<.001	<.001	<.001	<.001
Change in percentage over 1 year, percentage points^g^	−30.5	−29.8	−31.4	−26.3	−27.3
**XR naltrexone**					
Estimated intercept					
Prepandemic^c^	0.02 (0.02 to 0.02)	0.03 (0.02 to 0.03)	0.02 (0.02 to 0.02)	0.02 (0.02 to 0.02)	0.006 (0.005 to 0.007)
Pandemic^d^	0.01 (0.01 to 0.02)	0.02 (0.02 to 0.02)	0.01 (0.01 to 0.02)	0.02 (0.02 to 0.02)	0.005 (0.004 to 0.005)
Before-to-after change in level (%)^f^	−0.003 (−15.0)	−0.004 (−13.3)	−0.003 (−15.0)	−0.003 (−15.0)	−0.001 (−16.7)
*P* value	<.001	<.001	<.001	<.001	.01
Estimated weekly trend (slope)					
Prepandemic^c^	0.00005 (0.00003 to 0.00007)	0.00006 (0.00003 to 0.00008)	0.00006 (0.00002 to 0.00009)	0.00007 (0.00004 to 0.0001)	0.00004 (0.000003 to 0.00007)
Pandemic^d^	0.000009 (−0.000001 to 0.00002)	0.000003 (−0.00001 to 0.00002)	0.00002 (−0.0000006 to 0.00004)	0.00001 (−0.000003 to 0.00003)	0.00001 (−0.000007 to 0.00003)
Before-to-after change in trend (ie, slope)	−0.00004	−0.00005	−0.00004	−0.00006	−0.00002
*P* value	<.001	.001	.05	.002	.2
Change in percentage over 1 year, percentage points^g^	−10.7	−9.9	−10.4	−15.6	−26.0

Abbreviation: XR, extended release.

^a^ The estimated weekly rate of patients with filled buprenorphine or XR naltrexone prescriptions for opioid use disorder per 1000 patients with any prescription drug claim are presented overall and by race and ethnicity before and after the transition week (ie, March 16-22, 2020) starting immediately after the national emergency declaration and depicting the onset of the pandemic. Weeks with national holidays (ie, November 26, 2019; December 24, 2019; and January 1, 2020) and the week of March 8, 2020, were excluded to smooth data.

^b^ Estimates are calculated from ordinary least squares regressions of the weekly rate of patients with filled buprenorphine or XR naltrexone prescriptions for opioid use disorder on weekly trends (interrupted time series analyses).

^c^ March 2-8, 2020.

^d^ March 16-22, 2020.

^e^ Differences in intercepts and slopes and their 95% CI were used to test before-to-after change.

^f^ The percentage change in intercepts was calculated based on the before-after change in level and prepandemic level.

^g^ The change in percentage over 1 year was the difference between the annual prepandemic trend (eg, total number of patients who filled buprenorphine prepandemic: 0.004 × 52/0.7 × 100 = 29.71%) and annual pandemic trend (eg, total number of patients who filled buprenorphine: −0.001 × 52/0.7 × 100 = −0.74%), implying an annualized pandemic change of −0.74% minus −29.71% = −30.5%.

**Figure.**
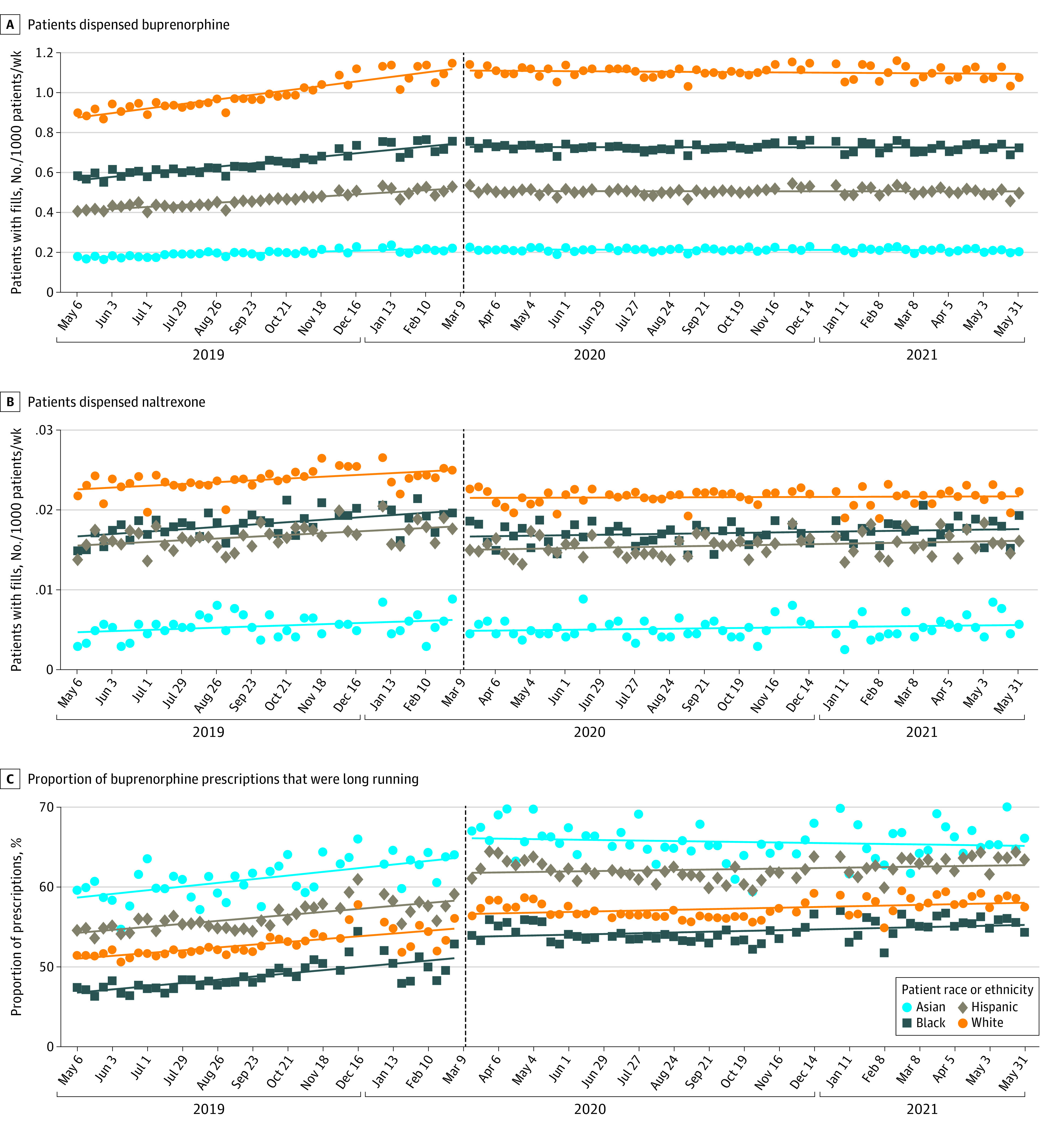
Estimated Weekly Prescription Fills by Race and Ethnicity Weekly estimated prescription fills are shown for buprenorphine and extended-release naltrexone from May 2019 to May 2021. Long-running buprenorphine prescriptions fills are those with 14 days or more of supply.

After the onset of the pandemic, significant decreases in estimated weekly rate of buprenorphine prescription fills per 1000 individuals (ie, intercept) were observed for Black patients (2.5%; *P* = .009), Hispanic patients (4.0%; *P* = .009), and Asian patients (4.0%; *P* = .04) but not White patients (1.8%; *P* = .15) (Table 1). We did not estimate decreases for the unspecified race or ethnicity category, although these individuals are included in the total column. However, despite level differences in outcomes, trends were similar for the full sample and subsamples with complete or missing race or ethnicity information for all outcomes throughout the study period (eFigure 1 in the Supplement). Thus, interrupted time series estimates, which consider changes in intercepts and trends within each sample, captured pandemic changes in each group. Estimated weekly XR naltrexone filled prescriptions per 1000 individuals decreased significantly overall (−15.0%; *P* < .001) and across racial and ethnic groups (ranging from −13.3% for White patients; *P* < .001 to −16.7% for Asian patients; *P* = .01) (Table 1). The change in annualized trends associated with the pandemic was estimated as the difference between the 1-year growth rate before and after pandemic onset. Over 1 year, the change in slopes amounted to a 30.5 percentage point decrease in the rate of buprenorphine filled prescriptions overall, 27.3 percentage points among Asian individuals, 26.3 percentage points among Black individuals, 31.4 percentage points among Hispanic individuals, and 29.8 percentage points among White individuals (all *P* < .001). Although the decrease was smaller for Black patients than White patients, the decrease in levels (significant only for racial and ethnic minority populations) meant that the overall net pandemic decrease (level plus trend change) was greater for racial and ethnic minority groups. For example, the net annualized pandemic decrease was 29.0% (95% CI, 34.2%-23.7%) among White patients and 32.7% (95% CI, 38.1%-27.3%) among Black patients. Similarly, the change in slope over 1 year equated to a 10.7 percentage point decrease in the rate of patients filling XR naltrexone prescriptions overall (*P* < .001), 15.6 percentage points among Black patients (*P* = .002), 10.4 percentage points among Hispanic patients (*P* = .04), and 9.9 percentage points among White patients (*P* = .001); the change in slope for Asian patients was not statistically significant.

Prior to the pandemic, the estimated proportion of weekly buprenorphine prescription fills that were longer term (ie, ≥14 days’ supply) was 51.9% (95%CI, 51.1%-52.6%), with some variation by patient race and ethnicity (63.8 [95% CI, 62.4%-65.3%] for Asian patients, 51.2% [95%CI, 50.4%-52.0%] for Black patients, 58.4% [95%CI, 57.6%-59.2%] for Hispanic patients, and 54.9% [95%CI, 54.2%-55.6%] for White patients) (Table 2). Moreover, this share of longer prescriptions was trending upward. The pandemic onset was associated with an immediate level increase in proportion of longer-duration buprenorphine prescription fills overall (2.0 percentage points; *P* < .001) and for all racial and ethnic groups (2.3 percentage points for Asian patients; *P* = .01; 2.5 percentage points for Black patients; *P* < .001; 3.3 percentage points for Hispanic patients; *P* < .001; and 1.7 percentage points for White patients; *P* < .001) (Table 2). However, considering trend decreases, our estimates imply that over a 1-year period the pandemic onset was associated with a 6.0 percentage point decrease in the trend for overall share of longer buprenorphine prescription fills and 9.8 percentage points for Asian patients, 8.1 percentage points for Black patients, 6.2 percentage points for Hispanic patients, and 6.6 percentage points for White patients (all *P* < .001) (Table 2).

**Table 2.** Changes in Estimated Percentage of Buprenorphine Prescription Fills That Were Long Running^a^

Variable	Filled prescriptions, % (95% CI)				
	Total	White patients	Hispanic patients	Black patients	Asian patients
Estimated intercept^b^					
Prepandemic^c^	51.9 (51.1 to 52.6)	54.9 (54.2 to 55.6)	58.4 (57.6 to 59.2)	51.2 (50.4 to 52.0)	63.8 (62.4 to 65.3)
Pandemic^d^	53.9 (53.3 to 54.5)	56.6 (56.0 to 57.1)	61.8 (61.2 to 62.4)	53.7 (53.1 to 54.3)	66.1 (65.1 to 67.2)
Before-to-after change in level, percentage points (%)^e^^,^^f^	2.0 (3.9)	1.7 (3.1)	3.3 (5.7)	2.5 (4.9)	2.3 (3.6)
*P* value	<.001	<.001	<.001	<.001	.01
Estimated weekly trend (slope)					
Prepandemic^c^	0.08 (0.05 to 0.1)	0.09 (0.06 to 0.1)	0.09 (0.07 to 0.1)	0.1 (0.07 to 0.1)	0.1 (0.06 to 0.2)
Pandemic^d^	0.02 (0.006 to 0.04)	0.02 (0.007 to 0.04)	0.02 (−0.001 to 0.03)	0.02 (0.007 to 0.04)	−0.02 (−0.04 to 0.01)
Before-to-after change in trend (slope)	−0.06	−0.07	−0.08	−0.08	−0.1
*P* value	<.001	<.001	<.001	<.001	<.001
Change in percentage over 1 year, percentage points^g^	−6.0	−6.6	−6.2	−8.1	−9.8

^a^ The estimated percentages of buprenorphine prescriptions for opioid use disorder that were long running (ie, with ≥14 days of supply) in a given week overall and by race and ethnicity before and after the transition week (ie, March 16-22, 2020) starting immediately after the national emergency declaration and depicting the onset of the pandemic are presented. Weeks with national holidays (ie, November 26, 2019; December 24, 2019; and January 1, 2020) and the week of March 8, 2020, were excluded to smooth the data.

^b^ Estimates are calculated from ordinary least squares regressions of the percentage of long-running buprenorphine prescription fills for opioid use disorder on weekly trends (the interrupted time series analyses).

^c^ March 2-8, 2020.

^d^ March 16-22, 2020.

^e^ Differences in intercepts and slopes and their 95% CI were used to test before-to-after change.

^f^ The percentage change in intercepts was calculated based on the before-after change in level and prepandemic level.

^g^ The change in percentage over 1 year was the difference between the annual prepandemic trend (eg, any patients who filled buprenorphine prescriptions: 0.08 × 52/51.9 × 100% = 8.0%) and annual pandemic trend (eg, any patients who filled buprenorphine prescriptions: 0.02 × 52/51.9 × 100% = 2.0%), implying an annualized pandemic change of 2.0% − 8.0% = −6.0 percentage points.

### Buprenorphine and XR Naltrexone Prescribing by Payer Type

For the 2 largest racial or ethnic groups (ie, White and Black patients), we further examined differential changes by payer type (Table 3; eFigure 2 in the Supplement). For White patients, the immediate onset of the pandemic was associated with level decreases in the estimated weekly rate of buprenorphine fills per 1000 individuals among Medicare (−3.5%; *P* = .004) and cash payers (−15.0%; P < .001), with a concurrent 3.3% increase (*P* = .02), in the estimated weekly rate of buprenorphine fills to the commercially insured White patient population (Table 3). For White patients, the pandemic onset was also associated with a decrease in the weekly buprenorphine trend across all payer categories (1-year decrease of 7.8 percentage points, 18.2 percentage points, 24.2 percentage points, and 50.3 percentage points for Medicaid, cash paying, Medicare, and commercially insured patients, respectively) (all *P* < .001).

**Table 3.** Changes in Estimated Prescription Fills by Payer Type^a^

Variable	Patients with fills, No. (95% CI), patients/1000 patients/wk							
	White patients				Black patients			
	Medicaid	Medicare	Private insurance	Cash	Medicaid	Medicare	Private insurance	Cash
**Buprenorphine**								
Estimated intercept^b^								
Prepandemic^c^	0.4 (0.4 to 0.4)	0.2 (0.2 to 0.2)	0.3 (0.3 to 0.4)	0.2 (0.2 to 0.2)	0.3 (0.3 to 0.3)	0.1 (0.1 to 0.1)	0.2 (0.2 to 0.2)	0.1 (0.1 to 0.1)
Pandemic^d^	0.4 (0.4 to 0.4)	0.2 (0.2 to 0.2)	0.4 (0.3 to 0.4)	0.2 (0.2 to 0.2)	0.3 (0.3 to 0.3)	0.1 (0.1 to 0.1)	0.2 (0.2 to 0.2)	0.10 (0.10 to 0.1)
Before-to-after change in level (%)^e^^,^^f^	−0.002 (−0.7)	−0.007 (−0.004)	0.01 (−0.02)	−0.03 (less than −0.001)	−0.008 (−0.05))	−0.010 (less than −0.001)	0.006 (−0.2)	−0.02 (less than −0.001)
*P* value	.67	.004	.02	<.001	.05	<.001	.18	<.001
Estimated weekly trend (slope)								
Prepandemic^c^	0.001 (0.001 to 0.002)	0.001 (0.001 to 0.001)	0.002 (0.002 to 0.003)	0.0009 (0.0007 to 0.001)	0.0010 (0.0007 to 0.001)	0.001 (0.0009 to 0.001)	0.002 (0.001 to 0.002)	0.0005 (0.0004 to 0.0007)
Pandemic^d^	0.0004 (0.0002 to 0.0006)	0.00007 (−0.000008 to 0.0002)	−0.0009 (−0.001 to −0.0007	0.0002 (0.0001 to 0.0003)	0.0004 (0.0003 to 0.0005)	−0.00003 (−0.00010 to 0.00005)	−0.0006 (−0.0007 to −0.0005)	0.0002 (0.0002 to 0.0003)
Before-to-after change in trend (ie, slope)	−0.0009	−0.001	−0.003	−0.0007	−0.0006	−0.001	−0.002	−0.0003
*P* value	<.001	<.001	<.001	<.001	<.001	<.001	<.001	<.001
Change in percentage over 1 year, percentage points^g^	−7.8	−24.2	−50.3	−18.2	−10.4	−53.6	−67.6	−15.6
**XR naltrexone**								
Estimated intercept								
Prepandemic^c^	0.01 (0.01 to 0.01)	0.003 (0.002 to 0.003)	0.008 (0.008 to 0.008)	0.001 (0.001 to 0.001)	0.01 (0.01 to 0.01)	0.003 (0.003 to 0.003)	0.005 (0.005 to 0.006)	0.0007 (0.0006 to 0.0008)
Pandemic^d^	0.01 (0.01 to 0.01)	0.002 (0.002 to 0.002)	0.007 (0.007 to 0.008)	0.0010 (0.0009 to 0.001)	0.009 (0.009 to 0.010)	0.002 (0.002 to 0.002)	0.004 (0.004 to 0.005)	0.0006 (0.0005 to 0.0007)
Before-to-after change in level (%)^e^^,^^f^	−0.002 (−20.0)	−0.0006 (−20.0)	−0.0008 (−10.0)	−0.0002 (−20.0)	−0.002 (−20.0)	−0.0007 (−23.3)	−0.0008 (−18.0)	−0.00009 (−12.9)
*P* value	<.001	<.001	<.001	.002	<.001	<.001	<.001	.28
Estimated weekly trend (slope)								
Prepandemic^c^	0.00003 (0.00001 to 0.00004)	0.000006 (0.0000008 to 0.00001)	0.00003 (0.00002 to 0.00004)	0.00001 (0.00001 to 0.00002)	0.00003 (0.000003 to 0.00005)	0.00002 (0.000007 to 0.00003)	0.00004 (0.00002 to 0.00005)	0.000006 (0.000001 to 0.00001)
Pandemic^d^	0.0000001 (−0.000008 to 0.000008)	0.000003 (0.0000007 to 0.000006)	0.000001 (−0.000005 to 0.000007)	0.000002 (−0.0000001 to 0.000004)	0.00002 (0.000006 to 0.00003)	0.000004 (−0.000001 to 0.000010)	−0.00001 (−0.00002 to −0.000003)	0.000002 (−0.0000009 to 0.000004)
Before-to-after change in trend (ie, slope)	−0.00003	−0.000002	−0.00003	−0.00001	−0.000007	−0.00001	−0.00005	−0.000004
*P* value	.001	.46	<.001	<.001	.62	.02	<.001	.12
Change in percentage over 1 year, percentage points^g^	−15.5	−5.2	−18.9	−41.6	−5.2	−27.7	−52.0	−29.7

Abbreviation: XR, extended release.

^a^ The estimated weekly numbers of White and Black patients with filled buprenorphine or XR naltrexone prescriptions for opioid use disorder by payer type per 1000 patients with any prescription drug claim before and after the transition week (ie, March 16-22, 2020) starting immediately after the national emergency declaration and depicting the onset of the pandemic are presented. Weeks with national holidays (ie, November 26, 2019; December 24, 2019; and January 1, 2020) and the week of March 8, 2020, were excluded to smooth data.

^b^ Estimates were calculated from ordinary least squares regressions of weekly number of White or Black patients with filled buprenorphine or XR naltrexone prescriptions for opioid use disorder on weekly trends (interrupted time series analyses).

^c^ March 2-8, 2020.

^d^ March 16-22, 2020.

^e^ Differences in intercepts and slopes and their 95% CI were used to test before-to-after change.

^f^ The percentage change in intercepts was calculated based on the before-after change in level and prepandemic level.

^g^ The change in percentage over 1 year was the difference between the annual prepandemic trend (eg, White patients paying with Medicaid who filled buprenorphine prescriptions: 0.001 × 52/0.4 × 100% = 13.0%) and annual pandemic trend (eg, White patients paying with Medicaid who filled buprenorphine prescriptions: 0.0004 × 52/0.4 × 100% = 5.2%), implying an annualized pandemic change of 5.2% − 13.0% = −7.8 percentage points.

Stratified by insurance type, the pandemic was associated with level and trend changes in weekly buprenorphine prescribing that were less favorable for Black than White patients within each insurance type, with significant changes. For Black Medicare- and cash-paying patients, prescribing levels decreased by 10.0% and by 20.0%, respectively (*P* < .001 for both) (Table 3), although not for those paying with Medicaid or commercial insurance. Among Black patients, the onset of the pandemic was associated with a larger decrease in the trend in weekly buprenorphine prescribing across all payer categories, implying 1-year decreases of 10.4 percentage points, 15.6 percentage points, 53.6 percentage points, and 67.6 percentage points for Medicaid, cash paying, Medicare, and commercially insured patients, respectively (*P* < .001 for all).

The level of estimated weekly XR naltrexone prescribing fills also significantly decreased after the pandemic onset (Table 3), with uniform immediate level decreases across nearly all payer types for White and Black patients (ranging from 10.0% for White patients paying via commercial insurance; *P* <.001 to 23.3% for Black patients paying via Medicare; *P* < .001). The exception was Black patients paying in cash (Table 3), for whom the level decrease in estimated weekly XR naltrexone prescription fills was not statistically significant. Changes in trends in estimated XR naltrexone prescription fills after pandemic onset were less uniform, decreasing by 15.5 percentage points for White patients paying via Medicaid (*P* = .001), 41.6 percentage points for White patients paying with cash (*P* < .001), 18.9 percentage points for White patients paying via commercial insurance (*P* < .001), 27.7 percentage points for Black patients paying via Medicare (*P* = .02), and 52.0 percentage points for Black patients paying via commercial insurance (*P* < .001).

In days-supply analyses among White patients, a significant level increase was observed in the share of longer buprenorphine prescriptions for Medicaid (1.9 percentage points; *P* < .001) and cash payment sources (5.1 percentage points; *P* < .001) (Table 4; eFigure 3 in the Supplement). For Black patients, the onset of the pandemic was associated with a similar level increase in the share of longer buprenorphine prescriptions for all insurance types except patients paying with private insurance (ranging from 1.5 percentage points for Medicare; *P* = .03 to 6.3 percentage points for cash; *P* < .001). Trend analyses presented a different picture; trends in the share of buprenorphine prescriptions with at least 14 days’ supply decreased significantly relative to prepandemic trends for White and Black patients across all payment sources, except Medicaid for White patients and cash for Black patients (Table 4).

**Table 4.** Changes in Estimated Percentage of Buprenorphine Prescription Fills That Were Long Running by Payer Type^a^

Variable	Filled prescriptions, % (95% CI)							
	White patients				Black patients			
	Medicaid	Medicare	Private insurance	Cash	Medicaid	Medicare	Private insurance	Cash
**Estimated percentage^b^**								
Prepandemic^c^	39.8 (38.9 to 40.7)	66.5 (65.6 to 67.4)	65.8 (64.6 to 67.0)	43.5 (42.8 to 44.2)	39.3 (38.4 to 40.2)	64.0 (62.9 to 65.1)	62.0 (60.5 to 63.6)	37.5 (36.4 to 38.6)
Pandemic^d^	41.7 (41.1 to 42.4)	67.0 (66.4 to 67.7)	64.5 (63.6 to 65.4)	48.6 (48.1 to 49.1)	42.8 (42.1 to 43.5)	65.5 (64.7 to 66.3)	60.7 (59.5 to 61.9)	43.7 (42.9 to 44.6)
Before-to-after change in level, percentage points (%)^e^^,^^f^	1.9 (4.8)	0.6 (0.9)	−1.3 (11.7)	5.1 (2.0)	3.5 (8.9)	1.5 (2.3)	−1.3 (16.8)	6.3 (2.1)
*P* value	<.001	.34	.10	<.001	<.001	.03	.17	<.001
**Estimated weekly trend (slope)**								
Prepandemic^c^	0.04 (0.01 to 0.08)	0.1 (0.09 to 0.2)	0.004 (−0.04 to 0.05)	0.1 (0.1 to 0.2)	0.06 (0.03 to 0.09)	0.1 (0.1 to 0.2)	−0.02 (−0.08 to 0.03)	0.10 (0.06 to 0.1)
Pandemic^d^	0.01 (−0.006 to 0.03)	−0.02 (−0.04 to 0.0009)	0.08 (0.05 to 0.1)	0.10 (0.09 to 0.1)	−0.003 (−0.02 to 0.01)	0.005 (−0.02 to 0.03)	0.09 (0.06 to 0.1)	0.1 (0.10 to 0.1)
Before-to-after change in trend (slope)	−0.03	−0.1	0.07	−0.03	−0.06	−0.1	0.1	0.02
*P* value	.08	<.001	.007	.02	.001	<.001	<.001	.40
Change in percentage over 1 year, percentage points^g^	−3.9	−9.4	0	6.0	−8.3	-7.7	0	9.2

^a^ The estimated percentages of buprenorphine prescriptions for opioid use disorder filled by White or Black patients that were long running (with ≥14 days of supply) in a given week by payer type before and after the transition week (ie, March 16-22, 2020) starting immediately after the national emergency declaration and depicting the onset of the pandemic are presented. Weeks with national holidays (ie, November 26, 2019; December 24, 2019; and January 1, 2020) and the week of March 8, 2020, were excluded to smooth the data.

^b^ Estimates were calculated from ordinary least squares regressions of the percentage of long-running buprenorphine prescription fills for opioid use disorder on weekly trends (the interrupted time series analyses).

^c^ March 2-8, 2020.

^d^ March 16-22, 2020.

^e^ Differences in intercepts and slopes and their 95% CI were used to test before-to-after change.

^f^ The percentage change in intercepts was calculated based on the before-after change in level and prepandemic level.

^g^ The change in percentage over 1 year was the difference between the annual prepandemic trend (eg, White patients paying with Medicaid: 0.04 × 52/39.8 × 100% = 5.2%) and annual pandemic trend (eg, White patients paying with Medicaid: 0.01 × 52/39.8 × 100% = 1.3%), implying an annualized pandemic change of 1.3% − 5.2% = −3.9 percentage points.

## Discussion

This cross-sectional study found that buprenorphine and XR naltrexone prescribing fills were trending upward prior to the COVID-19 pandemic, with substantially higher rates for buprenorphine than XR naltrexone. This steady increase was interrupted by the pandemic; prescribing plateaued across all racial and ethnic groups, with immediate decreases and complete flattening of the prior upward trend. Results on prescribing levels implied 30.5% and 10.7% reductions, respectively, for buprenorphine and XR naltrexone. We also found that racial and ethnic minority groups had larger losses in level of buprenorphine and XR naltrexone pharmacy dispensing fills during the pandemic. Specifically, after March 2020, the decrease in level of buprenorphine prescription fills was statistically significant only for racial and ethnic minority groups (Asian, Black, Hispanic patients).

This greater disruption in filled buprenorphine and naltrexone prescriptions among racial and ethnic minority groups is concerning given that rates of fatal overdoses among these subpopulations have increased rapidly.^21,22^ For instance, deaths from synthetic opioids other than methadone increased 18-fold among Black patients and 12-fold among Hispanic patients from 2013 to 2017 compared with a 9-fold increase among White patients.^6,29^ These disparities are associated with multiple structural inequities, including inadequate community supply and geographic maldistribution of clinicians prescribing MOUD in minority populations with greater overdose rates; financial barriers, such as out-of-pocket cost burdens; prior authorization requirements that may have disproportionate impacts^30^; and missed opportunities to initiate treatment at touch points, such as overdose treatment episodes.^12,23,31,32^ These inequities in access may have been associated with the disproportionate adverse impact of the pandemic among minority populations, who experienced higher rates of COVID-19 infection, hospitalization, and mortality, as well as unemployment and other adverse outcomes. During the pandemic, payers, regulators, and clinicians and health care institutions introduced more flexibility for telehealth prescribing of MOUD, but economically disadvantaged populations with more limited access to technology may have experienced barriers to these modalities. These potential inequities warrant further research specifically addressing clinician distribution, access to technology, and other barriers that may have been associated with disadvantages among minority populations.

Our results also suggest important issues for further research on disparities by insurance coverage, given that initiatives to reduce barriers varied across payer types. Our findings on disparate trends by payer type suggest that pandemic outcomes also varied by payer type. For example, buprenorphine prescription fills among the commercially insured White population increased in level by 3.3% at the onset of the pandemic, suggesting that the White commercially insured patient population may have had resources to better cope with disruptions in health care access during pandemic-related shutdowns. It is also noteworthy that there were no level decreases in buprenorphine prescription fills among Medicaid patients in White or Black populations. This finding suggests that Medicaid programs may have provided greater safety nets to alleviate pandemic-related losses of income and health insurance coverage compared with other payer types. Furthermore, the finding suggests that policy initiatives in Medicaid programs, such as encouragement of telehealth flexibility and, in some cases, distribution of smartphones, may have buffered some disparate impacts of the pandemic on access. Further studies should focus on Medicaid access initiatives across states and the role of Medicaid expansion.

The onset of the pandemic was associated with more uniform 13.3% to 16.7% decreases in dispensing fills of XR naltrexone for OUD and a concurrent near flattening of growth rates (−9.9 percentage points to −15.6 percentage points decreases in trends over 1 year) across all racial and ethnic groups. These results are consistent with high out-of-pocket costs of XR naltrexone.^11^ Moreover, unlike buprenorphine, XR naltrexone had decreases in prescription fills that were similar across patient race and ethnic groups. However, XR naltrexone continues to make up a much smaller share of MOUD prescribing.

Our analysis suggests that the COVID-19 pandemic was associated with intensification of prior racial and ethnic disparities in MOUD treatment access.^12^ High medication costs may be an important financial barrier for low-income minority populations, especially in states without Medicaid expansion.^12^

Our findings may have implications for future federal, state, and local policies to curb the continuing overdose epidemic and narrow health disparities in the US. Prior work found that buprenorphine prescribers were primarily located in White neighborhoods^18^; therefore, buprenorphine for OUD was still largely unavailable to patients who are members of racial ethnic minority groups. This access barrier was even more problematic during the pandemic, given that buprenorphine may be administered with minimal contact and was therefore preferable to limit the transmission of the virus. Policies like eliminating buprenorphine waiver requirements and providing necessary training to physicians and advanced practitioners in primary care settings may have helped improve availability. However, the high cost of buprenorphine and XR naltrexone for OUD may be prohibitive for patients with low incomes, decreasing retention and adherence.^33^ The COVID-19 pandemic may have been associated with exacerbated financial barriers to buprenorphine and naltrexone for OUD among racial and ethnic minority groups, who have experienced greater economic losses during the pandemic.

### Limitations

Our study has several limitations. First, the study spans a relatively short period and we cannot precisely identify treatment initiation vs continuation to disentangle discontinued treatment vs decreased initiations. Second, we captured only aggregate nationwide changes. Future research should assess changes by states, which may have differed in their pandemic severity, pandemic mitigation policies, pandemic MOUD policies, and prepandemic policies, including Medicaid expansion status. Third, much like prior work using retail pharmacy claims data,^20^ we were unable to track dispensing of MOUD via opioid treatment programs given that these medications do not flow through retail pharmacies. Fourth, for a considerable share of patients (46.5%-47.4%), race and ethnicity were unspecified.

## Conclusions

In this cross-sectional study, we documented decreases in the number of filled prescriptions for buprenorphine and XR naltrexone, the 2 most commonly prescribed MOUD, during the 15 months after the pandemic onset. To our knowledge, this is the first study to quantify disproportionate outcomes associated with pandemic-related disruptions in buprenorphine and XR naltrexone use among racial and ethnic minority populations already at increased risk of barriers to access. Our analysis used 92% of US retail pharmacy claims between May 2019 and June 2021, with race and ethnicity data spanning all insurer types and uninsured individuals, finding that buprenorphine and XR naltrexone prescription fills were trending upward prior to the pandemic. However, the pandemic onset was associated with immediate changes in levels and longer-term trend changes in prescribing, with plateaus to prepandemic growth in filled prescriptions. Moreover, these decreases were larger across insurance types for racial and ethnic minority populations, which already had lower filled prescription rates prior to the pandemic and experienced disproportionately higher COVID-19 and opioid overdose risks during the pandemic. Overall, we found that COVID-19 may have been associated with negative outcomes among numerous policy efforts aimed at curbing the opioid epidemic over the past decade, along with worsened disparities in buprenorphine and XR naltrexone pharmacy dispensing fills among racial and ethnic minority populations.

Overall, our results suggest the pressing need for research that more clearly defines use barriers for particular subpopulations to MOUD in major payer systems, assesses state-level MOUD initiatives; more clearly identifies the role of geographical maldistribution of providers, and closely assesses equity in access to telehealth and the role of policies that may decrease these disparities. These policies may include smartphone distribution to individuals with low incomes needing telehealth access; the potential disparate impact of prior authorization requirements; deployment of peer navigator programs for individuals at increased risk of barriers to access; elimination of barriers, such as special training requirements for buprenorphine prescribing; and encouragement of clinicians who are key touch points for individuals at increased risk to prescribe MOUD.

## Notes

Supplement. **eTable.** Weekly Shares of Individuals With Filled Prescriptions by Race and Ethnicity and Payer Type **eFigure 1.** Changes in No. of Unique Individuals With Prescription Fills and Percentage of Buprenorphine Fills That Were Long Running by Availability of Race and Ethnicity Data **eFigure 2.** Changes in Patients With Prescription Fills by Race and Ethnicity and Payer Type **eFigure 3.** Changes in Percentage of Prescriptions That Were Long Running by Race and Ethnicity and Payer Type
