# Errors in Tables and Results

**DOI:** 10.1001/jamanetworkopen.2022.23561

**Published:** 2022-07-05

In the Original Investigation titled “Racial and Ethnic Disparities in Buprenorphine and Extended-Release Naltrexone Filled Prescriptions During the COVID-19 Pandemic,”^1^ published June 1, 2022, there were errors in before-to-after changes in level percentages for buprenorphine in Table 3 and Table 4, errors in before-to-after changes in trend percentages over 1 year in Table 4, and a misstatement of 2 subgroups in the Results in reference to Table 4 data on trends in share of buprenorphine prescriptions with at least 14 days’ supply. This article has been corrected.^1^
