# Supplementary material for: Racial and Ethnic Disparities in Buprenorphine and Extended-Release Naltrexone Filled Prescriptions During the COVID-19 Pandemic
Source: JAMA Netw Open. 2022 Jun 1;5(6):e2214765. doi: 10.1001/jamanetworkopen.2022.14765 (PMC9161014; doi:10.1001/jamanetworkopen.2022.14765)
Supplement: Supplement. — eTable. Weekly Shares of Individuals With Filled Prescriptions by Race and Ethnicity and Payer Type eFigure 1. Changes in No. of Unique Individuals With Prescription Fills and Percentage of Buprenorphine Fills That Were Long Running by Availability of Race and Ethnicity Data eFigure 2. Changes in Patients With Prescription Fills by Race and Ethnicity and Payer Type eFigure 3. Changes in Percentage of Prescriptions That Were Long Running by Race and Ethnicity and Payer Type [file jamanetwopen-e2214765-s001.pdf]

Supplemental Online Content

Nguyen T, Ziedan E, Simon K, et al. Racial and ethnic disparities in buprenorphine and extended-release naltrexone filled prescriptions during the COVID-19 pandemic. *JAMA Netw Open*. 2022;5(6):e2214765. doi:10.1001/jamanetworkopen.2022.14765

eTable. Weekly Shares of Individuals With Filled Prescriptions by Race and Ethnicity and Payer Type
eFigure 1. Changes in No. of Unique Individuals With Prescription Fills and Percentage of Buprenorphine Fills That Were Long Running by Availability of Race and Ethnicity Data
eFigure 2. Changes in Patients With Prescription Fills by Race and Ethnicity and Payer Type
eFigure 3. Changes in Percentage of Prescriptions That Were Long Running by Race and Ethnicity and Payer Type

This supplemental material has been provided by the authors to give readers additional information about their work.

© 2022 Nguyen T et al. *JAMA Network Open*.

eTable. Weekly Shares of Individuals With Filled Prescriptions by Race and Ethnicity and Payer Type

	Buprenorphine patients				Vivitrol patients			
	Any weeks (N=101 weeks)	Pre-pandemic mean (SD)	Pandemic mean (SD)	P values	Any weeks (N=101 weeks)	Pre-pandemic mean (SD)	Pandemic mean (SD)	P values
Share of Asian patients	0.3 (0.0)	0.3 (0.0)	0.3 (0.0)	<0.001	0.3 (0.1)	0.3 (0.1)	0.3 (0.1)	0.44
Share of Black patients	5.5 (0.1)	5.5 (0.0)	5.5 (0.1)	0.98	6.2 (0.4)	6.2 (0.4)	6.1 (0.4)	0.18
Share of Hispanic patients	3.0 (0.0)	3.0 (0.0)	3.1 (0.0)	<0.001	4.5 (0.3)	4.5 (0.4)	4.5 (0.2)	0.48
Share of white patients	46.6 (0.3)	46.4 (0.1)	46.8 (0.2)	<0.001	44.5 (0.9)	44.2 (0.9)	45.0 (0.8)	<0.001
Share of other patients	44.7 (0.3)	44.9 (0.1)	44.4 (0.2)	<0.001	44.5 (0.8)	44.7 (0.8)	44.1 (0.7)	<0.001
Share of Asian Medicaid patients	30.6 (2.3)	30.0 (2.1)	31.5 (2.2)	<0.001	41.7 (12.6)	39.9 (12.3)	44.3 (12.6)	0.087
Share of Asian Medicare patients	13.6 (1.7)	14.0 (1.5)	12.9 (1.8)	<0.001	10.1 (8.0)	10.2 (7.9)	9.9 (8.4)	0.85
Share of Asian private patients	31.4 (2.5)	32.4 (2.3)	30.0 (2.1)	<0.001	38.0 (11.5)	38.6 (12.0)	37.1 (10.8)	0.52
Share of Asian cash patients	17.4 (1.8)	16.6 (1.5)	18.5 (1.6)	<0.001	5.0 (6.1)	5.3 (6.0)	4.5 (6.4)	0.52
Share of Black Medicaid patients	41.3 (1.5)	41.0 (1.3)	41.7 (1.7)	0.016	58.4 (3.2)	57.9 (3.2)	59.0 (3.1)	0.10
Share of Black Medicare patients	17.4 (0.6)	17.6 (0.4)	17.3 (0.8)	0.014	13.8 (2.1)	13.7 (2.3)	13.9 (1.9)	0.68
Share of Black private patients	24.3 (2.0)	24.9 (1.8)	23.5 (1.9)	<0.001	24.4 (3.2)	24.4 (3.3)	24.4 (3.1)	0.94
Share of Black cash patients	15.0 (0.9)	14.5 (0.8)	15.7 (0.5)	<0.001	3.5 (1.2)	3.9 (1.2)	3.0 (0.9)	<0.001
Share of Hispanic Medicaid patients	39.3 (1.2)	39.0 (0.7)	39.8 (1.5)	<0.001	57.9 (2.9)	58.1 (3.0)	57.6 (2.8)	0.36
Share of Hispanic Medicare patients	15.5 (0.6)	15.6 (0.4)	15.3 (0.7)	0.005	11.2 (2.4)	10.3 (2.3)	12.5 (1.9)	<0.001

© 2022 Nguyen T et al. *JAMA Network Open.*

Share of Hispanic private patients	27.3 (1.1)	27.5 (1.0)	27.0 (1.2)	0.007	26.7 (3.1)	27.1 (2.8)	26.1 (3.4)	0.11
Share of Hispanic cash patients	14.1 (0.7)	13.8 (0.7)	14.5 (0.6)	<0.001	3.3 (1.5)	3.8 (1.5)	2.6 (1.1)	<0.001
Share of white Medicaid patients	35.6 (1.0)	35.7 (1.0)	35.5 (1.0)	0.19	49.8 (1.8)	48.8 (1.4)	51.2 (1.0)	<0.001
Share of white Medicare patients	14.4 (0.6)	14.8 (0.3)	14.0 (0.6)	<0.001	10.1 (0.8)	9.9 (0.8)	10.4 (0.7)	0.002
Share of white private patients	29.3 (1.6)	29.8 (1.6)	28.7 (1.4)	<0.001	32.9 (1.8)	34.0 (1.3)	31.3 (1.4)	<0.001
Share of white cash patients	16.0 (1.0)	15.3 (0.7)	17.0 (0.4)	<0.001	4.3 (0.9)	4.8 (0.5)	3.5 (0.9)	<0.001

© 2022 Nguyen T et al. *JAMA Network Open.*

eFigure 1. Changes in No. of Unique Individuals With Prescription Fills and Percentage of Buprenorphine Fills That Were Long Running by Availability of Race and Ethnicity Data

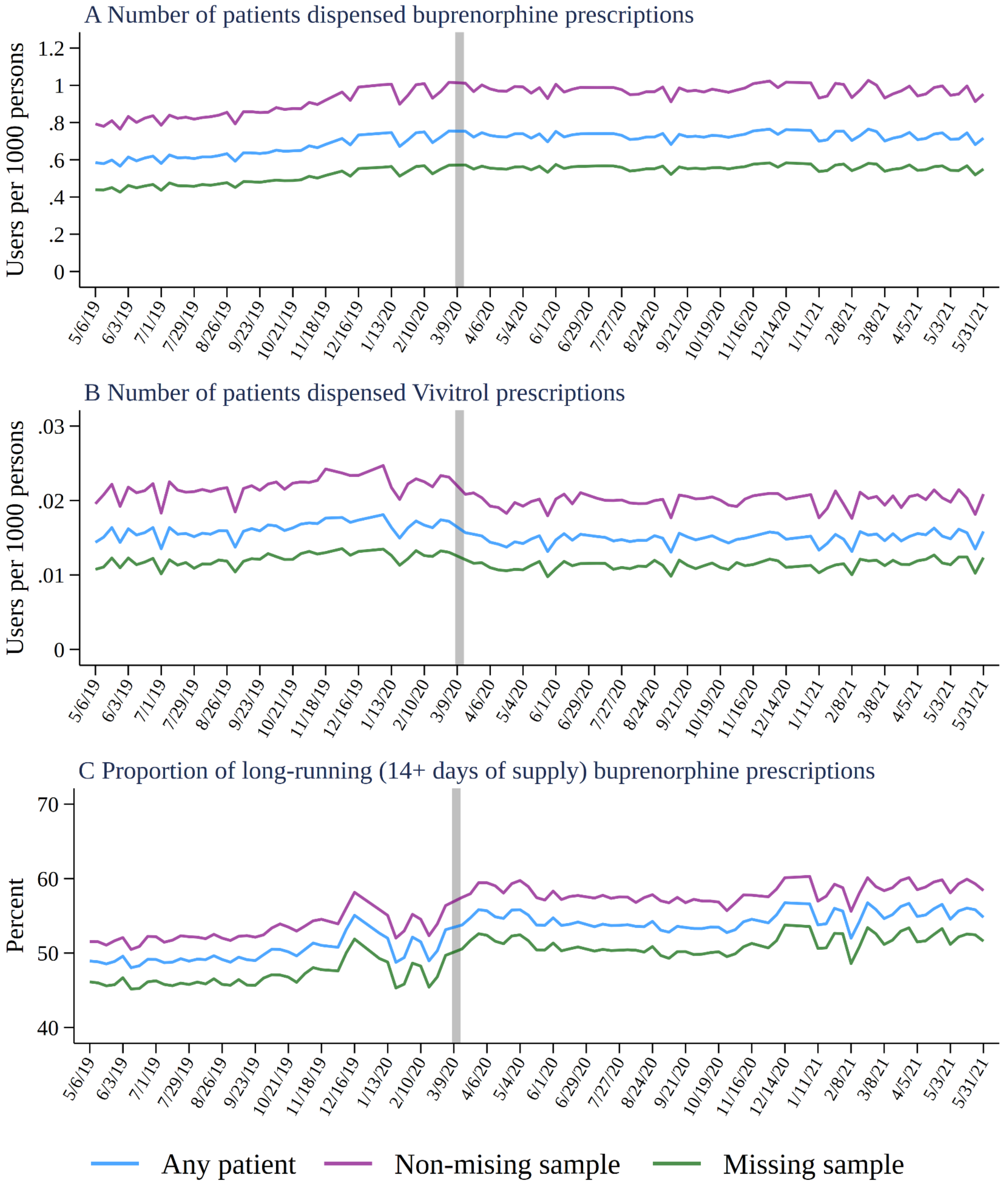

© 2022 Nguyen T et al. *JAMA Network Open.*

eFigure 2. Changes in Patients With Prescription Fills by Race and Ethnicity and Payer Type

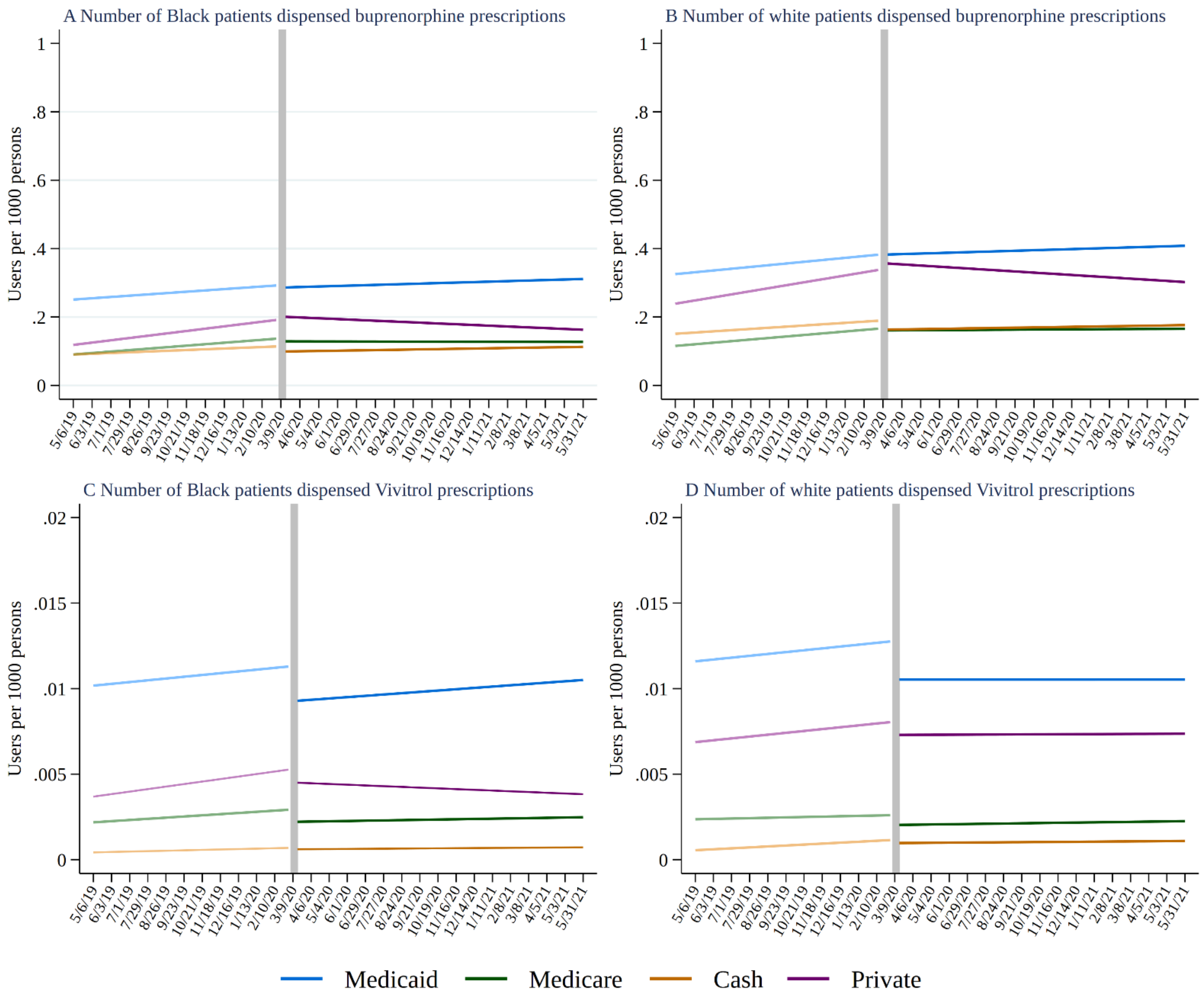

A and C: number of unique Black patients filling a prescription for bupreorphine (or Vivitrol) for OUD in a given week, per 1000 patients with any prescription drug claim in that week (lines represent regression adjusted values). B and D: number of unique white patients filling a prescription for bupreorphine (or Vivitrol) for OUD in a given week, per 1000 patients with any prescription drug claim in that week (lines represent regression adjusted values). The gray vertical line designates the transition week (i.e., March 16-22, 2020) starting immediately following the National Emergency Declaration and depicting the onset of the pandemic. We excluded several weeks with national holidays: November 26, 2019, December 24, 2019, and January 1, 2020, to smooth the data.

© 2022 Nguyen T et al. *JAMA Network Open.*

eFigure 3. Changes in Percentage of Prescriptions That Were Long Running by Race and Ethnicity and Payer Type

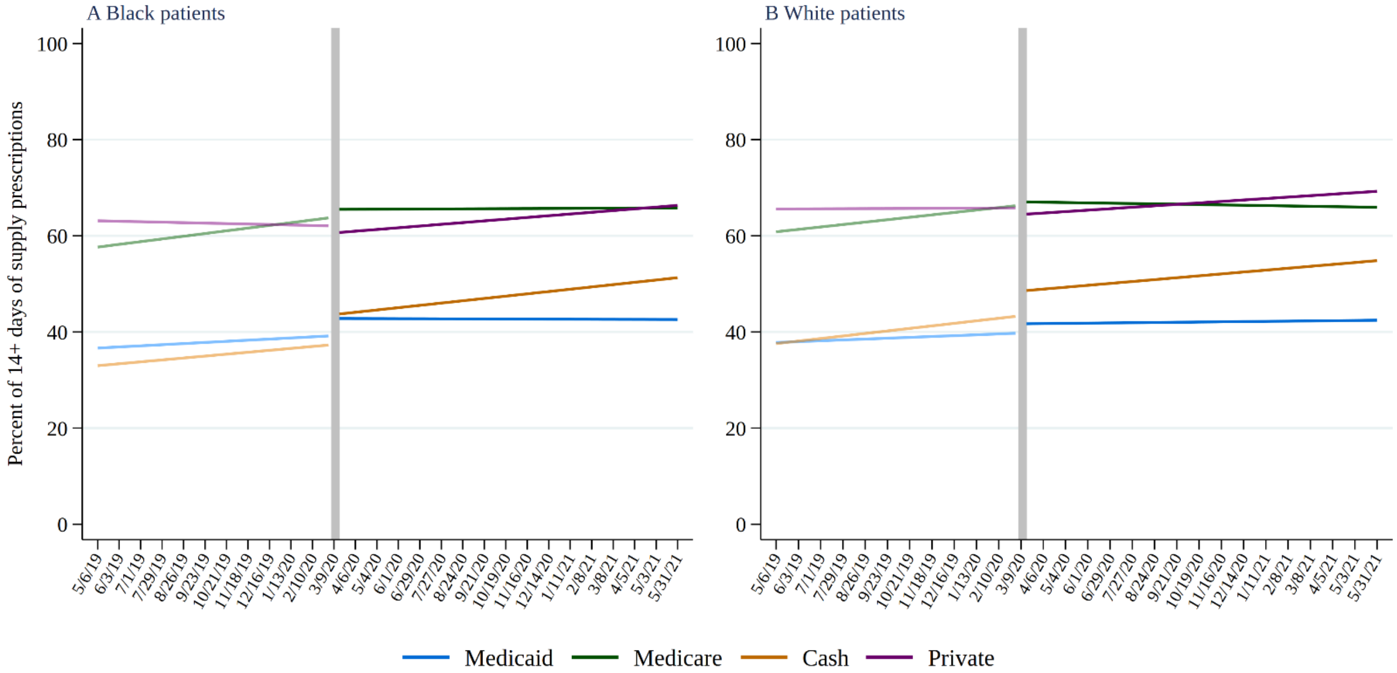

March 16-22, 2020) starting immediately following the National Emergency Declaration and depicting the onset of the pandemic. We excluded several weeks with national holidays: November 26, 2019, December 24, 2019, and January 1, 2020, to smooth the data. The gray vertical line designates the transition week (i.e., March 16-22, 2020) starting immediately following the National Emergency Declaration and depicting the onset of the pandemic. We excluded several weeks with national holidays: November 26, 2019, December 24, 2019, and January 1, 2020, to smooth the data.

© 2022 Nguyen T et al. *JAMA Network Open*.
